# Does Local Coherence Lead to Targeted Regressions and Illusions of Grammaticality?

**DOI:** 10.1162/opmi_a_00041

**Published:** 2021-07-06

**Authors:** Dario Paape, Shravan Vasishth, Ralf Engbert

**Affiliations:** Department of Linguistics, University of Potsdam, Potsdam, Germany; Department of Linguistics, University of Potsdam, Potsdam, Germany; Department of Psychology, University of Potsdam, Potsdam, Germany

**Keywords:** local coherence, eye tracking, grammaticality judgments, targeted regressions

## Abstract

Local coherence effects arise when the human sentence processor is temporarily misled by a locally grammatical but globally ungrammatical analysis (*The coach smiled at the player tossed a frisbee by the opposing team*). It has been suggested that such effects occur either because sentence processing occurs in a bottom-up, self-organized manner rather than under constant grammatical supervision, or because local coherence can disrupt processing due to readers maintaining uncertainty about previous input. We report the results of an eye-tracking study in which subjects read German grammatical and ungrammatical sentences that either contained a locally coherent substring or not and gave binary grammaticality judgments. In our data, local coherence affected on-line processing immediately at the point of the manipulation. There was, however, no indication that local coherence led to illusions of grammaticality (a prediction of self-organization), and only weak, inconclusive support for local coherence leading to targeted regressions to critical context words (a prediction of the uncertain-input approach). We discuss implications for self-organized and noisy-channel models of local coherence.

## INTRODUCTION

Ever since their discovery by Tabor et al. (2004), local coherence effects have posed an interesting conundrum for standard theories of human sentence processing. The sentence in (1a) has only one correct parse, in which the noun phrase *the player* is modified by a reduced relative clause and the agent of the verb *toss* remains unrealized. The exact same parse is available for sentence (1b), where the relative clause is unreduced, and for (1c), which replaces the passive participle with that of a semantically similar verb, *throw*.

(1)  a. The coach smiled at the player tossed a frisbee by the opposing team.
  b. The coach smiled at the player who was tossed a frisbee by the opposing team.
  c. The coach smiled at the player thrown a frisbee by the opposing team.

In a self-paced reading experiment Tabor et al. (2004) found that the words *tossed*/*thrown a frisbee* took longer to process in (1a) compared to (1b) and (1c). They replicated the effect in a second self-paced reading study and in a grammaticality judgment study, where (1a) was more likely to be rejected than (1b) and (1c). This pattern is consistent with the idea that in (1a), the human sentence processor is led astray due to the substring *the player tossed a frisbee* being a possible active main clause, even though it appears at a position in the sentence where such an analysis is ruled out by grammar. Tabor et al. (2004) argue that such local coherence effects may be best explained by parsing models that relax the principle of grammatical self-consistency, which requires that the parser only considers globally legal analyses at every point during the parse. A class of models that do not assume self-consistency are self-organized parsing models such as SOPARSE (Tabor & Hutchins, 2004) and Unification Space (Vosse & Kempen, 2000). In these models, lexical items bring with them small pieces of syntactic structure that may freely combine at any point during processing and attempt to form a globally well-formed tree structure (Smith, 2018; Smith et al., 2018; Villata & Franck, 2020). If multiple locally well-formed attachments compete with each other, processing difficulty arises. Slowed reading in locally coherent structures is naturally accounted for by this kind of model as the underlined local analysis in (1a) competes with the globally correct analysis. If the conflict between the two cannot be resolved, parsing failure may result.

An entirely different explanation of local coherence effects, the noisy-channel model, was proposed by Levy (2008b) and tested by Levy et al. (2009). The model rests on the assumption that readers maintain some uncertainty about the words they have read, and may consider the possibility that they did not read the word *at* in (1a), but rather the word *and* or the word *as*, or that *at* may have been a typo. The main intuition is that readers may change their assumptions about the identity of previously read words when subsequent input does not easily fit with the current parse. For instance, in (1a), changing *at* to *as* only requires substitution of one letter and results in the possibility of a high-probability alternative parse in which *tossed* is analyzed as an active verb (*The coach smiled as the player tossed a frisbee*). Levy (2008b) assumes that large changes in the reader’s belief about the previous input result in slowed processing and, if possible, in regressive saccades, due to lingering uncertainty about the actual identity of the critical word *at*. Given that the alternative parse is not available in (1b, 1c), no revision of beliefs is triggered at *tossed/thrown*, and the difference between conditions is thus accounted for.

Given the assumed link between uncertainty about previous input and overt regressions in the noisy-channel model, data from eye tracking during reading are potentially informative when it comes to dissociating the two proposed explanations.^1^ Research on temporarily ambiguous garden-path sentences has yielded some evidence that readers engage in targeted rereading during reanalysis (Frazier & Rayner, 1982; Meseguer et al., 2002), though less systematically than had initially been believed (Mitchell et al., 2008; von der Malsburg & Vasishth, 2011, 2013). For local coherence sentences, Levy et al. (2009) found that readers made more regressive eye movements and spent more time rereading earlier parts of the sentence in (1a) compared to (1c) after having first fixated the verb *tossed*/*thrown*. In (1a), readers were also more likely to refixate the preposition *at* than in (1c). This latter finding is consistent with readers trying to resolve the conflict between *the player* having been attached as the object of *smiled at* and the locally coherent attachment of *the player* as the subject of the would-be active verb *tossed*. Crucially, Levy et al. (2009) did not observe the same effect when the preposition *at* was replaced with *toward*. They argue that the asymmetry is due to *toward* being less easily confused with *as* or *and*, thus supporting the Levy (2008b) account over the self-organized parsing account of Tabor et al. (2004).

In another set of two eye-tracking studies, Christianson et al. (2017) investigated targeted regressions in locally coherent structures and classic garden-path structures. In one of their experiments, they observed additional rereading of the region containing the preposition for local coherence sentences. However, regressions to precritical regions also occurred in control sentences similar to (1b), so it is not clear whether they were triggered by local coherence per se. Christianson et al. (2017) conclude that participants in their study largely used rereading to confirm their interpretation of the previous input, as opposed to revising their analysis. Furthermore, given that comprehension accuracy was low even when rereading had occurred, Christianson et al. (2017) suggest that participants engaged in superficial or “good enough” processing (e.g., Christianson et al., 2001; Ferreira et al., 2002), possibly adopting syntactic representations of the input. They speculate that “rereading may signal… a cobbling together of several smaller interpretations based on substrings that are not fully integrated into a single global structure” (p. 23). The notion of a “cobbled-together” analysis is compatible with a self-organization mechanism, where partial parses compete with each other. Depending on the implementation, the system may settle in a state in which either two globally incompatible attachments are active at the same time, or in a state in which there is no chain of attachments that spans the entire input (Smith, 2018). However, given that Christianson et al.’s (2017) interpretation of the reading patterns is speculative and based solely on the low comprehension accuracy observed in their study, it is not clear which kinds of representations subjects ended up with, or how they were derived.

In summary, evidence from eye-tracking studies does not decisively favor either the self-organization or the uncertainty-based view of local coherence effects. This is especially true given that regressions occur relatively rarely in the eye-tracking record, constituting only 10–15% of all saccades (Rayner, 2009), and therefore relatively many observations are needed to draw reliable conclusions about targeted regressions in particular. One aim of the current study is to investigate whether the pattern of targeted regressions discovered by Levy et al. (2009) can be replicated with a different kind of local coherence in a different language, namely, German. We deviate from previous studies in that each sentence is followed by a binary grammaticality judgment as opposed to a comprehension question. In theory, such a task may lead to more regressions compared to asking comprehension questions, as formal features of the sentence become more important than meaning-related ones. We also test a prediction derived from self-organized parsing, namely, that local coherence may result in illusions of grammaticality: If a given sentence is globally ungrammatical but strongly locally coherent, the local parse may act as the syntactic and semantic representation of the entire input, leading to incorrect positive grammaticality judgments. Smith (2018) reports simulation results from a self-organized parsing system that support this prediction: Given a specific set of modeling assumptions, the system settled on the local parse in 18 out of 100 trials for the English materials of Tabor et al. (2004).

Our study uses sentences such as (2).

(2)  Man erfuhr später, dass …
  *One learned later that*
  … einer der Spitzel enttarnte Informanten warnten.
   *one of.the snitches exposed informants warned.pl*

Verb-final word order is mandatory in German embedded clauses headed by the complementizer *dass*, “that,” unless the verb takes a sentential object. The embedded clause contains an adjectival participle form such as *enttarnte*, “exposed,” that is superficially identical to the third-person singular active form of the same verb. This results in a locally coherent subject-verb-object (SVO) structure in which the syntactic object *enttarnte Informanten*, “exposed informants,” could instead be analyzed as a verb-object sequence (see also Konieczny et al., 2009; Paape & Vasishth, 2016). The sentence in (2) is globally ungrammatical, as the number feature the subject noun phrase *einer der Spitzel*, “one of the snitches,” does not match the number feature of the verb *warnten*, “warned.pl.” When subjects read the sentence and judge its grammaticality, they may experience processing difficulty because there are two competing syntactic analyses of the input that are somewhat attractive but are nevertheless both incorrect. The two competing analyses are shown in (3) below. Note that when *enttarnte*, “exposed,” is analyzed as a verb, the final verb *warnten*, “warned.pl,” is left without an attachment site.

(3) 
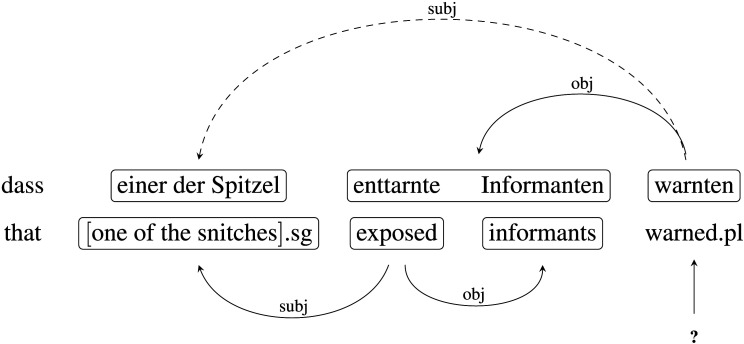

We now turn to our experimental study.

## MATERIALS AND METHODS

### Participants

Seventy-seven German native speakers were recruited from the local student population at the University of Potsdam.^2^ All had normal or corrected-to-normal eyesight. They were paid 15€ as compensation for their participation.

### Design and Stimuli

We preregistered our design and analysis, without theory-specific predictions, at https://osf.io/ersxn. Our experiment followed a 2 × 2 design adapted from Paape and Vasishth (2016). We manipulated the factors’ grammaticality (grammatical versus ungrammatical) and local coherence (locally coherent versus not locally coherent). Grammaticality was manipulated by having the final verb of the embedded clause correctly agree with the subject in number or not. The would-be SOV structure is only locally coherent when the would-be active verb *enttarnte*, “exposed,” agrees in number with the subject, and therefore local coherence can be manipulated via the subject’s number feature. Thirty-two experimental items were created according to the scheme shown in (4). Diamonds indicate region-of-interest boundaries in the example stimulus.

(4)  **Preamble**
  Man erfuhr später, ⋄ dass ⋄ …
  *One learned later that*
  **Grammatical, Locally coherent**
  … einer der Spitzel ⋄ enttarnte Informanten ⋄ […] ⋄ warnte.
   *one of.the snitches exposed informants  warned.sg*
  **Grammatical, Not locally coherent**
  … einige der Spitzel ⋄ enttarnte Informanten ⋄ […] ⋄ warnten.
   *some of.the snitches exposed informants  warned.pl*
  **Ungrammatical, Locally coherent**
  … einer der Spitzel ⋄ enttarnte Informanten ⋄ […] ⋄ warnten.
   *one of.the snitches exposed informants  warned.pl*
  **Ungrammatical, Not locally coherent**
  … einige der Spitzel ⋄ enttarnte Informanten ⋄ […] ⋄ warnte.
   *some of.the snitches exposed informants  warned.sg*

The elided […] in the example was occupied by a prepositional phrase, in this case *mit raffinierten Tricks*, “with subtle ploys.” The prepositional phrase was always three words long and semantically compatible with both the global and the locally coherent analyses (“expose with subtle ploys”/“warn with subtle ploys”).

### Procedure

After giving informed consent, participants read the stimulus sentences and indicated after each trial whether the sentence had been grammatical or not. The experimental sentences were presented according to a Latin-square design. They were mixed with 96 filler sentences containing different kinds of grammatical violations, including agreement violations, case-marking errors, as well as missing or added words. Overall, half of the sentences in the experiment were ungrammatical. Presentation order was randomized at runtime. Participants were seated at a distance of 60 cm from the presentation screen, which had a resolution of 1920 × 1080 pixels. Sentences were presented in 16 pt black Courier font on white background. Eye movements from the right eye were recorded with an EyeLink 1000 Tower Mount eye-tracking system at a sampling frequency of 1,000 Hz. Recalibration using a 9-point grid was carried out every 15 trials or as needed. Drift correction was performed every six trials. Each trial started with the presentation of a fixation cross at the position of the initial letter of the sentence. After participants fixated the cross, the sentence was presented. Participants indicated that they wanted to continue to the grammaticality judgment by looking in the lower right corner of the screen, then gave their judgment by pressing one of two buttons on a VPixx RESPONSEPixx response box. The experiment started with six practice sentences to familiarize participants with the procedure.

### Data Analysis

Due to technical failures, no eye tracking data were collected for two participants. No accuracy data were collected for six participants due to a programming error. Data from one additional participant were removed because the participant failed to reach 50% accuracy on grammaticality judgments for the experimental items. All analyses of eye-tracking measures are thus based on the data from 74 participants, while analyses of question responses are based on data from 68 participants.^3^ Fixations and saccades were computed from the raw data using the algorithm of Engbert and Mergenthaler (2006). Fixations shorter than 20 ms were discarded and reading time measures for the subject, object, post-object, and verb regions were computed using the em2 package (Logačev & Vasishth, 2013) in R (R Core Team, 2019). In an additional step that we had not preregistered, for each reading time measure, data points below 80 ms were also discarded, as 80 ms have been estimated as the minimum amount of time within which linguistic information can influence oculomotor control (Altmann, 2011). Statistical analyses were performed by fitting fully hierarchical linear mixed models using the brms package (Bürkner, 2017). We analyzed log-transformed first-pass reading times, regression-path durations and total reading times, in addition to log-transformed judgment response times.^4^ The analysis of judgment response times included an additional shift parameter to account for motor execution time (Rouder, 2005). Fully hierarchical logistic regression models were used to analyze response accuracy and regression probabilities. Both experimental factors were sum-coded, with grammatical and locally coherent coded as 1 and ungrammatical and not locally coherent coded as −1. Normal(0, 10) priors were used for intercepts and Normal(0, 1) priors^5^ were used for the slopes across all analyses. LKJ priors (Lewandowski et al., 2009) were used for the correlation matrices, with the *ν* parameter set to 2, making correlations near ±1 a priori less likely. Four sampling chains with 2,000 iterations each were run for each model. The first 1,000 samples were discarded as warmup. We treat effects as reliable if 95% of the posterior probability are either above or below zero. Code and full posterior plots for all analyses, along with our experimental data and analysis code are available at https://osf.io/f6ntk.

## PREDICTIONS

Previous findings by Levy et al. (2009), Christianson et al. (2017), and Müller and Konieczny (2019) indicate that processing disruption caused by local coherence should become visible in first-pass reading times and regression-path durations. In our materials, the local coherence begins at the object region, so that the disruption is predicted to occur in this region, either because an error signal is triggered (Levy, 2008b) or because the locally coherent analysis competes with the global one (Tabor et al., 2004).

### Self-Organized Parsing Models

There exists, to our knowledge, no implemented model that spells out the assumed connections between a self-organized parser and the eye movement control system. Accordingly, any experimental outcome in which the eye movement record shows an indication of processing difficulty—longer reading times and/or regressions—in the conditions where self-organized parsing predicts increased competition is, in principle, compatible with the theory.

The predictions of the self-organized model depend on when the system settles on an analysis. If the system does *not* settle before the entire input has been processed, readers may experience prolonged processing difficulty until the final region, due to competition between the local and global analyses. Alternatively, if the competition is short-lived and the system settles on an analysis already at the object region, no prolonged difficulty is expected. Predictions for the sentence-final region also depend on whether the system has settled on an analysis: If the parser has settled on the locally coherent parse, ungrammaticality of the global analysis presumably should not matter, and processing difficulty should thus not differ between grammatical and ungrammatical sentences. This prediction assumes that the parser can settle on sufficiently well-formed (“harmonious”) structures even if not all words in the sentence have been integrated into the structure, as in the implementation of Smith (2018). However, given that the local analysis does not constitute a grammatical analysis of the entire input string, processing difficulty should nevertheless be higher than in grammatical sentences. By contrast, if the system has not settled on an analysis, competition should be greater between the local and global analyses when the global analysis is ungrammatical, because the two analyses will be closer to each other in well-formedness. In grammatical sentences, on the other hand, the globally correct analysis should outcompete the local one more quickly. Under both scenarios, a statistical interaction between local coherence and grammaticality is predicted in the final region.

Regarding the off-line judgments, if readers sometimes settle on the locally coherent analysis in ungrammatical sentences, they may experience illusions of grammaticality, leading to ungrammatical, locally coherent sentences being incorrectly judged as grammatical. Nevertheless, due to the lower harmony of the local coherence structure, there should be fewer positive grammaticality judgments than for grammatical sentences. By contrast, if readers are merely temporarily confused by the local coherence but do not adopt the incorrect parse, there should be no illusions of grammaticality. Konieczny (2005) found that participants took longer to notice ungrammaticality in locally coherent versus not locally coherent sentences, so we expect to find the same pattern in our study. Depending on when readers finish evaluating the sentence’s grammaticality, the effect may either appear in the final region of the sentence or in judgment response times.

### Noisy-Channel Model

Unlike self-organized parsing models, the noisy-channel model has no way of settling on ungrammatical structures, but is bound by grammatical self-consistency. Thus, the only scenario in which illusions of grammaticality could arise for ungrammatical, locally coherent sentences is one in which participants come to doubt the previous input and then fail to verify their revised belief about previous words’ identity through rereading. Essentially, in such trials the participant would come to believe that they are reading a main clause, and ignore the evidence to the contrary. Importantly, illusions of grammaticality should only arise if readers also ignore the sentence-final verb, because its presence is a strong indication that a main clause analysis is not tenable, even in ungrammatical sentences. Given that the noisy-channel model as formulated by Levy (2008b) only accounts for changes to previous but not to future input, it is thus unlikely that illusions of grammaticality should occur at all.

In the noisy-channel model, an error signal is raised when readers experience changes in their belief about previous input. The larger the change in belief, the larger the error signal. However, the strength of the error signal also depends on how easily the veridical input string can be edited into a “near-neighbor” string, such as *at* to *as*. The more distant the two strings are, the smaller the change in belief, given that smaller corruptions of the input in the noisy channel are more likely than larger corruptions. The model allows for deletions, insertions and substitutions of characters, and the penalty to the belief change incurred is proportional to the Levensthein distance between the veridical string and the transformed string.^6^ For our materials, there is only one set of transformations that we can think of that would license the local parse: Editing out the complementizer, adding a colon^7^ and capitalizing the first letter of the subject noun phrase, which turns the subordinate clause into a main clause with SVO word order, as shown in (5).

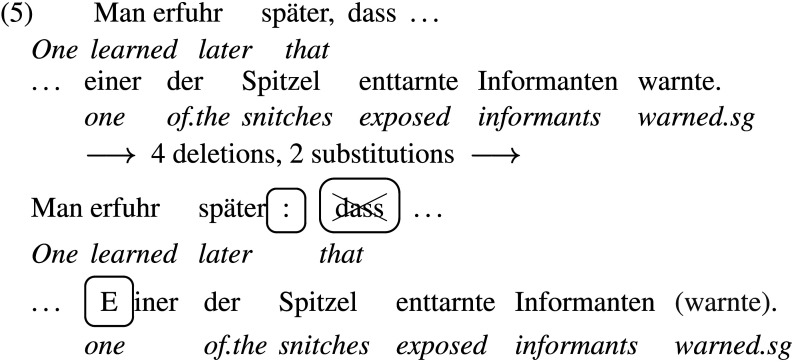

The edit distance here is twice as large as that assumed for the largest edits in the English materials, such as inserting *who* between *player* and *tossed*.^8^ The strength of the error signal also depends on whether *enttarnte*, “exposed,” is much more likely to appear as a finite verb in the edited main clause than as an adjective in the unedited subordinate clause. According to our seach in the TIGER treebank (Brants et al., 2002), the conditional probability of encountering a determiner-less, adjective-modified object noun phrase after a complementizer and a subject noun phrase (NP) is about 1.2%. By comparison, the conditional probability of encountering a finite verb directly after a colon and a subject NP is about 25%. In English, using the numbers obtained by Levy (2008b), the conditional probability of encountering a reduced relative clause after an NP is about 1.5%, compared to a 91% probability of encountering a finite verb after a subject NP. The local parse given the necessary edits is thus about 60 times more likely in the English materials and about 21 times more likely in the German materials. Based on the increased edit distance and the smaller change in conditional probability, the error signal should thus be much weaker in the German materials.

The hallmark prediction of the noisy-channel approach that was tested by Levy et al. (2009) is that readers make targeted regressions to previous words whose identity they have come to doubt. For the investigation of targeted regressions, the em2 package allows the computation of so-called conditional rereading measures, that is, measures that are contingent on a certain region in the sentence having been fixated prior to the rereading of an earlier region. Fixations on the “target” region are registered until the “source” region is passed to the right. We compute conditional rereading probabilities for three theoretically interesting target and source regions in the sentence and make predictions as shown in Table 1.

**Table 1.** Predictions for conditional rereading probabilities derived from the noisy-channel model. > indicates higher probability.

	**Source**	**Target**	**Prediction**
I	Object	Complementizer	Locally coherent > Not locally coherent
	*exposed informants*	*that*	
II	End of sentence	Object	Locally coherent > Not locally coherent
	*warned*	*exposed informants*	
III	End of sentence	Subject	Ungrammatical > Grammatical
	*warned*	*One/some of the snitches*	

Prediction I arises straightforwardly under the noisy-channel view, because reading the locally coherent object region may cause readers to check whether they are, in fact, reading a subordinate clause headed by *dass*, “that,” in which case the locally coherent parse is disallowed, in analogy to rereading the problematic word *at* in the English stimuli. Prediction II arises because encountering a verb at the end of the sentence is again clear evidence that the locally coherent region cannot also contain a verb, which may trigger confirmatory rereading. This prediction presupposes that the error signal triggered by the locally coherent adjective does not always immediately lead to regressions, but that readers may wait until further input is processed. Prediction III arises because encountering a verb with incorrect number marking could trigger uncertainty as to what the number feature of the subject phrase was, triggering confirmatory rereading.

## RESULTS

**Figure 1.**
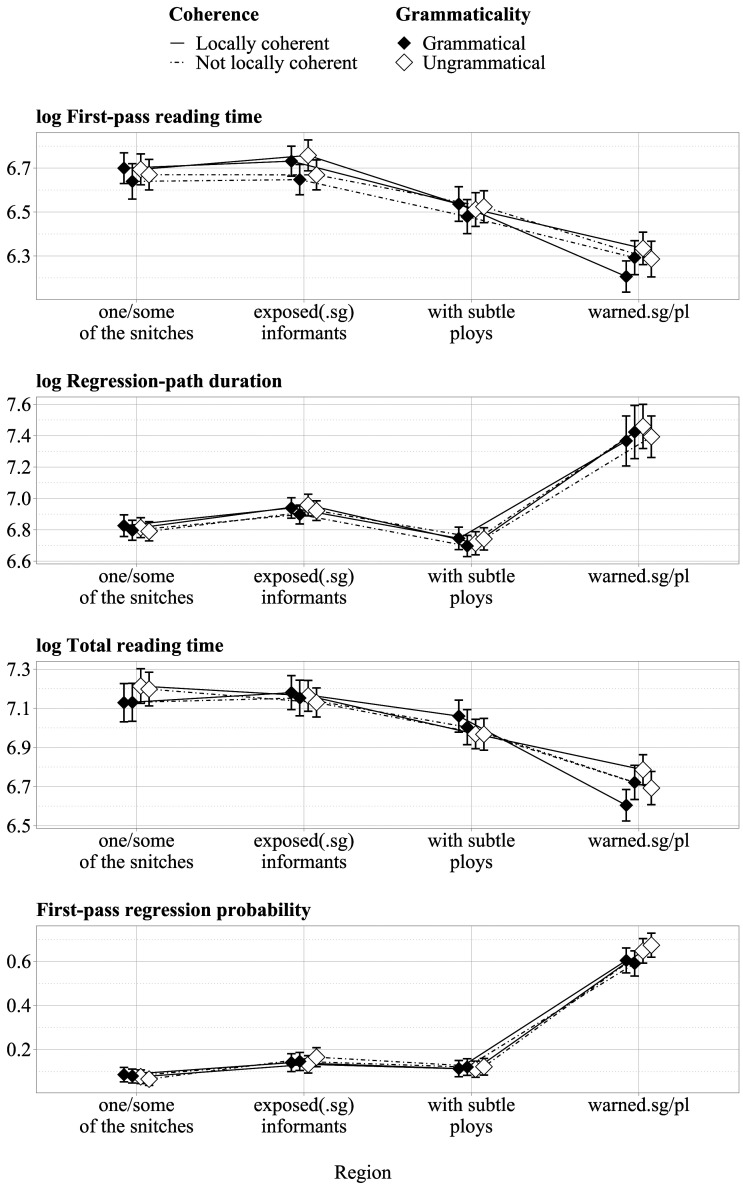
**Reading measures by region of interest and condition.** Error bars show 95% confidence intervals based on participant means.

Table 2 shows grammaticality judgment accuracy and mean response times by condition. Figure 1 shows reading measures by condition and region of interest.

**Table 2.** Grammaticality judgment accuracy and mean response times by condition.

**Grammaticality**	**Local coherence**	**p(correct)**	$\overset{ˉ}{RT}$ **(ms)**
Grammatical	Not locally coherent	91% [87%, 94%]	662 [576, 749]
Grammatical	Locally coherent	90% [86%, 94%]	654 [576, 733]
Ungrammatical	Not locally coherent	84% [80%, 89%]	616 [551, 681]
Ungrammatical	Locally coherent	87% [83%, 91%]	677 [576, 778]

### Judgment Response Times and Accuracy

There was no indication that the experimental manipulations affected judgment response times, but response accuracy was lower for ungrammatical compared to grammatical sentences ($\hat{\Delta }$ = −4%, CrI [credible interval]: [−7%, 0%]).

### Subject Region (one/some of the snitches)

Total reading times in the subject region were longer in ungrammatical compared to grammatical sentences ($\hat{\Delta }$ = 105 ms, CrI: [51 ms, 160 ms]).^9^

### Object Region (exposed informants)

First-pass reading times in the object region were increased in locally coherent compared to not locally coherent sentences ($\hat{\Delta }$ = 73 ms, CrI: [34 ms, 111 ms]). Regression-path durations in the object region were also longer in locally coherent compared to not locally coherent sentences ($\hat{\Delta }$ = 38 ms, CrI: [−3 ms, 80 ms]).

### Post-Object Region (with subtle ploys)

There was an interaction between grammaticality and local coherence at the post-object region ($\hat{\Delta }$ = −33 ms, CrI: [−66 ms, 0 ms]), mainly due to longer regression-path durations in locally coherent, grammatical sentences ($\hat{\Delta }$ = 41 ms, CrI: [−4 ms, 88 ms]). As the grammaticality of a given sentence is only determined in the sentence-final region, we assume that the pattern is either spurious or due to parafoveal preview. Total reading times for the post-object region where shorter in ungrammatical than in grammatical sentences ($\hat{\Delta }$ = −77 ms, CrI: [−135 ms, −20 ms]).

### Final Region (warned)

In the sentence-final region, first-pass reading times were longer in ungrammatical compared to grammatical sentences ($\hat{\Delta }$ = 35 ms, CrI: [9 ms, 62 ms]). There was also an interaction between grammaticality and local coherence ($\hat{\Delta }$ = 34 ms, CrI: [1 ms, 69 ms]), mainly due to shorter first-pass reading times in locally coherent, grammatical sentences ($\hat{\Delta }$ = −45 ms, CrI: [−84 ms, −7 ms]). There were more first-pass regressions from the sentence-final region in ungrammatical compared to grammatical sentences ($\hat{\Delta }$ = 0.07, CrI: [0.02, 0.12]). Regression-path durations in the sentence-final region showed the same interaction as first-pass reading times ($\hat{\Delta }$ = 113 ms, CrI: [−14 ms, 238 ms]), mainly due to shorter regression paths in locally coherent, grammatical sentences ($\hat{\Delta }$ = −115 ms, CrI: [−268 ms, 37 ms]). Total reading times in this region were longer for ungrammatical compared to grammatical sentences ($\hat{\Delta }$ = 69 ms, CrI: [30 ms, 108 ms]). Again, there was an interaction ($\hat{\Delta }$ = 88 ms, CrI: [48 ms, 129 ms]), due to local coherence leading to shorter total reading times in grammatical sentences ($\hat{\Delta }$ = −103 ms, CrI: [−151 ms, −55 ms]), but longer total reading times in ungrammatical sentences ($\hat{\Delta }$ = 69 ms, CrI: [30 ms, 107 ms]).

### Conditional Refixations

**Figure 2.**
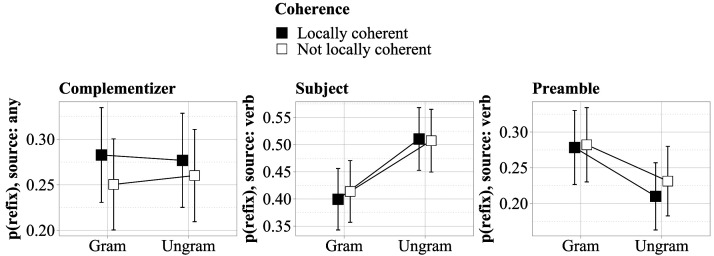
**Left: Refixations on the complementizer *dass*, “that,” after reading the locally coherent region, by condition. Middle: Refixations on the subject region after reading the sentence-final verb, by condition. Right: Refixations on the preamble region after reading the sentence-final verb, by condition.** Error bars show 95% confidence intervals based on participant means. Note the different scales.

In ungrammatical sentences, regression paths starting at the final verb were more likely to include the subject region ($\hat{\Delta }$ = 15%, CrI: [9%, 21%]) but less likely to include the sentence preamble ($\hat{\Delta }$ = −5%, CrI: [−9%, 0%]) compared to grammatical sentences. There was no indication of the other refixation patterns predicted by the noisy-channel approach.^10^ However, we also conducted an exploratory analysis of all regression paths starting at or after the object region, coding trials with at least one regression from any postsubject region versus trials with no regressions. In this analysis, there was some weak indication that the complementizer *dass*, “that,” was fixated more often in the locally coherent conditions ($\hat{\Delta }$ = 3%, CrI: [−1%, 7%]). The data underlying these analyses are shown in Figure 2.

## DISCUSSION

Local coherence led to increased first-pass reading times and regression-path durations in the object region (*enttarnte Informanten*, “exposed informants”), where the local parse became available, in line with findings by Bicknell et al. (2009), Levy et al. (2009), Christianson et al. (2017), and, more recently, by Müller and Konieczny (2019). There was some weak indication of local coherence triggering targeted regressions, as predicted by the noisy-channel model: While there was no indication that the local coherence immediately triggered regressions to the complementizer *dass*, “that,” regression paths starting at any point from the object phrase onward were more likely to include the complementizer by about 3% (CrI: [−1%, 7%]) in the locally coherent conditions. The effect is small and its credible interval includes zero, despite our sample being almost twice as large as that of Levy et al. (2009) and the paradigm being relatively efficient at triggering regressions: Overall, at least one interregion regression was made on 79% of trials for experimental sentences (CrI: [73%, 85%]), and the mean number of refixated regions per trial for these items was 2.3 (CrI: [2, 2.6]). Our paradigm was also, in principle, able to trigger targeted rereading. This is supported by the observation of more refixations of the subject region (15%, CrI: [9%, 21%]) and fewer refixations of the sentence preamble (−5%, CrI: [−9%, 0%]) in ungrammatical sentences. Participants presumably targeted the subject region to check whether the sentence was indeed ungrammatical. The finding is in line with the noisy-channel model, but could also be captured by other models, assuming that there is a strong link between attention and gaze location.

There is a question of whether a 3% increase in refixations of the complementizer provides convincing support for the noisy-channel model. Bayes factors can be used to quantify the evidence in favor of such an effect over the evidence in favor of the null hypothesis, which is that there is no effect of local coherence on refixations of the complementizer. We computed Bayes factors using bridge sampling (Meng & Wong, 1996). Given that vague priors lead to bias in favor of the null model (Lee & Wagenmakers, 2014), we also computed Bayes factors for two narrower priors, as shown in Table 3.

**Table 3.** Bayes factors (BF) for the effect of local coherence on refixations of the complementizer under different priors. A half-normal distribution is a normal distribution that is left-truncated at zero, thus restricting the prior to positive values. BF values >1 indicate evidence in favor of the null hypothesis.

Prior	Prior 95% CrI	BF_01_
Normal(0, 1)	[−62%, 62%]	7.14
Normal(0, 0.1)	[−7%, 7%]	1.05
Half-Normal(0.1)	[0%, 8%]	0.49

Assuming that previous results by Levy et al. (2009), who observed an increase of about 4% in refixations of the critical preposition, generalize to our German construction, assuming a small effect with a positive sign seems reasonable. On the other hand, if one does not subscribe to the noisy-channel model, other accounts of local coherence effects may predict fixations to be drawn *away* from the complementizer to other regions of the sentence, such as the beginning of the sentence or the locally coherent region, suggesting a wider prior. As expected, the null hypothesis is strongly favored under a wide prior, with the evidence tipping in favor of the alternative hypothesis under the narrowest prior. Nevertheless, the evidence in favor of the alternative hypothesis is still “weak” or “anecdotal” under this prior (Jeffreys, 1939/1998; Raftery, 1995). Our findings are thus *compatible* with the predictions of the noisy-channel model under a broad construal, but cannot be taken to directly support it, given that the analysis was exploratory in nature. Further confirmatory research is clearly needed.

Our findings are also compatible with the notion that rereading is largely confirmatory in local coherence structures (Christianson et al., 2017; Levy et al., 2009). For instance, there is no indication in our data that local coherence resulted in a complete revision of readers’ beliefs about the previous input: If readers had sometimes come to believe that they were reading a main clause, the sentence-final verb should have been highly surprising on such trials. Contrary to this possible prediction, there was no main effect of local coherence in any measure for the final region of the sentence; in fact, the numerical tendency for each of the three reading-time measures points in the opposite direction. This is broadly consistent with a version of the model in which readers always act on and eventually overcome uncertainty by “checking” previous input (overtly or covertly) when given the opportunity.^11^

There is no indication in our data that the availability of a locally coherent subparse leads to illusions of grammaticality in otherwise ungrammatical sentences. The prediction of self-organized processing models that the local analysis may outcompete the global one in ungrammatical sentences was thus not borne out in our stimuli. The null result is also in line with the findings of Konieczny (2005), who also found no indication of local coherence affecting the accuracy of grammatical error detection in German, albeit using a different type of structure. While we found no effects of the experimental manipulations on judgment response times, the pattern seen in total reading times in the sentence-final region is compatible with Konieczny’s (2005) finding that ungrammatical sentences take longer to reject in the presence of local coherence.

### Do Grammaticality and Local Coherence Interact? Investigating a Confound

The only robust interaction between the local coherence and grammaticality manipulations appeared across measures in the final region of the sentence, where agreement of the lexical verb with the sentential subject was either correct or not. The effect of local coherence in ungrammatical sentences is compatible with competition between the ungrammatical global analysis and the locally coherent analysis, as predicted by self-organized sentence processing. On the other hand, the speedup due to local coherence in grammatical sentences is unexpected. Readers may have suppressed the locally coherent parse during first-pass reading of the object region, forming a strong prediction about the upcoming verb’s number feature. In grammatical sentences, the prediction is borne out because verb carries the expected number feature, and processing is faster the locally coherent condition. In ungrammatical sentences, the prediction is not borne out and processing is slower in the locally coherent condition due to increased surprisal (Hale, 2001; Levy, 2008a).

**Figure 3.**
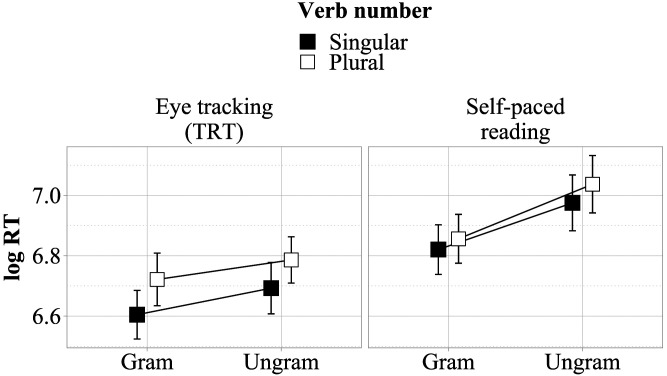
**Comparison of reading times at the sentence-final region between eye-tracking and self-paced reading experiments.** Error bars show 95% confidence intervals based on participant means.

However, this speculative explanation is called into question by the fact that our study confounded grammaticality and local coherence with the number feature of the sentence-final verb: The two slowest conditions in total reading times had plural verbs while the two faster conditions had singular verbs. Recoding the experimental factors accordingly, the grammaticality × local coherence interaction turns into a main effect of verb plurality (see following).^12^ Plural verbs may be more difficult to process than singular verbs because they are orthographically longer and morphologically more complex, or because plural number is more marked than singular number (e.g., Eberhard, 1997). In order to investigate this possibility, we conducted a web-based self-paced reading experiment with 69 participants that used the same materials (including the same fillers) and the same task as our eye-tracking study, but removed all locally coherent adjectives and replaced them with either definite articles, quantifiers, or unambiguous adjectives.^13^ The reading time results for the sentence-final verb are compatible with verb plurality being the source of the interaction: Reading times were slower for ungrammatical sentences ($\hat{\Delta }$ = 174 ms, CrI: [122 ms, 226 ms]) and also for sentences with plural verbs ($\hat{\Delta }$ = 51 ms, CrI: [10 ms, 91 ms]), resulting in a pattern that qualitatively matches that found in the eye-tracking study, as shown in Figure 3.

These additional results cast heavy doubt on the interpretation of the reading patterns observed in the main experiment as being due to an interaction between local coherence and grammaticality. While such an interaction may underlyingly be present, it would need to be tested more thoroughly in a design without the number confound.

### Self-Organized Processing and Noisy-Channel Models—The Broader Picture

When interpreting the current findings in relation to the larger theoretical picture, one also needs to keep in mind that the strength of local coherence effects may depend heavily on the type of structure used. Garden-path effects are known to differ in magnitude depending on the kind of ambiguity used (e.g., Sturt et al., 1999), with differences between ambiguity types going beyond what differences in predictability can account for (van Schijndel & Linzen, 2018). In this context, recall that Tabor et al. (2004) found more rejections of sentences containing English reduced relative clauses in the presence of local coherence, despite the fact that the sentences’ actual grammaticality remained unchanged. It could thus be argued that the self-organized approach would have predicted illusions of *ungrammaticality* rather than grammaticality for our sentences. Neither illusion was observed in our data. However, unlike our stimuli, which are perfectly natural for German readers, reduced relative clauses are known to be troublesome for English readers (Bever, 1970) and have been argued to be of dubious grammaticality under some conditions (McKoon & Ratcliff, 2003). Given these differences, a self-organized sentence processing model with the right parameter settings may be able to account for all the available data from both English and German. As the self-organized approach to sentence processing is still relatively young and implementation details differ between iterations, it remains to be seen whether a consensus model will emerge anytime soon. One key challenge is to explain the data of Levy et al. (2009), which, besides showing some evidence for targeted regressions, also suggest a role of orthographic or phonological similarity to “neighboring” words, as in the case of *at* versus *as*. The self-organized model of Kukona et al. (2014), which includes word recognition in addition to parsing and has been used to model fixation patterns in visual world experiments, could conceivably be extended to explain these patterns.

For the noisy-channel model of Levy (2008b), there are also considerable degrees of freedom with regard to the assumed link between error signal strength and eye movements, as well as possible effects of task manipulations on the model’s noise parameter, which influences the edit penalties. As our experiment contained ungrammatical sentences, participants may have inferred a higher level of noise in our stimuli compared to the studies of Tabor et al. (2004) and Levy et al. (2009) (see also Gibson et al., 2013). Relatedly, a more recent version of the noisy-channel account (Futrell et al., 2020) has been argued to account for illusions of grammaticality in complex English sentences, so that evidence for such illusions in locally coherent sentences, had we found any, would not have decisively favored the self-organized approach. However, as the account of Futrell et al. (2020) rests on readers completely forgetting about earlier parts of the sentence, it is unclear whether it can also plausibly account for illusions in short, simple sentences. Overall, collecting more high-quality data from different languages, using different experimental paradigms and different structures, and deriving testable predictions from implemented processing models will help to further disentangle the different existing accounts of local coherence effects.

## ACKNOWLEDGMENTS

The authors would like to thank Daniela Mertzen, Maximilian Rabe, and the audience at AMLaP 2019 in Moscow for fruitful discussions. Further thanks go to Sarah Risse and Hans Trukenbrod for assistance with the implementation of the experiment.

## FUNDING INFORMATION

SV, Deutsche Forschungsgemeinschaft (https://dx.doi.org/10.13039/501100001659), Award ID: 317633480. RE, Deutsche Forschungsgemeinschaft (https://dx.doi.org/10.13039/501100001659), Award ID: 317633480.

## AUTHOR CONTRIBUTIONS

DP: Conceptualization: Equal; Data curation: Lead; Formal analysis: Lead; Investigation: Lead; Methodology: Equal; Project administration: Supporting; Software: Supporting; Writing – original draft: Lead; Writing – review & editing: Equal. SV: Conceptualization: Equal; Funding acquisition: Lead; Methodology: Equal; Project administration: Lead; Resources: Supporting; Software: Supporting; Supervision: Lead; Writing – review & editing: Equal. RE: Conceptualization: Supporting; Funding acquisition: Lead; Methodology: Equal; Project administration: Lead; Resources: Lead; Software: Supporting; Supervision: Lead; Writing – review & editing: Equal.

## Notes

^1^ Note that we do not provide an exhaustive review of theories about local coherence effects that have been proposed in the literature. Other accounts include those of Gibson (2006), Bicknell et al. (2009), Morgan et al. (2010), and Hale (2011). See Paape and Vasishth (2016) for discussion.^2^ We had planned to test 80 participants, but remained three participants short of our goal due to project constraints and participant recruitment issues.^3^ As stated in the preregistration, all participants completed an operation-span test as a measure of working memory capacity prior to the experiment. These data were collected mainly for use in later stages of the project, where we intend to build a computational model that accounts for a variety of sentence-processing phenomena. We had no specific predictions regarding interactions between memory capacity and local coherence, and entering the memory measure into our current analyses yielded no informative interactions with the experimental manipulations, so that we refrain from reporting the results here.^4^ First-pass reading time is the sum of all fixation durations from first entering a region from the left prior to leaving it in any direction. Regression-path duration, also called go-past time, is first-pass reading time plus the sum of all fixations on earlier regions before passing the region to the right. Total reading time is the sum of all fixations in a region over the entire trial.^5^ We deviated from the preregistered Normal(0, 5) priors here because sampling is faster for more narrow priors and effects are not realistically expected to be larger than 2 log ms.^6^ Assuming that all edit operations are equally likely, it is irrelevant for the edit distance measure whether the veridical or the nonveridical sentence is taken as the “source” representation: From the reader’s point of view, the perceived sentence may appear like a corruption of an original, intended sentence—this is the idea behind the noisy-channel model—but from the experimenter’s perspective, the reader’s inferred representation is a corruption of the actual presented sentence. For reasons of expository ease, we present the experimenter’s perspective.^7^ See Levy (2011) for another application of the noisy-channel model that includes punctuation.^8^ Note that the comparison rests on the assumption by Levy (2008b) that edits are character-based. If edits are instead assumed to be word-based (Gibson et al., 2013), the contrast between our materials and the English examples is somewhat less stark.^9^ There was also some indication that first-pass reading times were increased in locally coherent sentences in the subject region, with the effect being just slightly below our predefined reliability criterion ($\hat{\Delta }$ = 28 ms, CrI: [−6 ms, 61 ms]). The difference may indicate a parafoveal preview effect, but a word-by-word analysis using successive differences contrasts reveals that conditions diverge at *einer*/*einige*, “one/some,” after which the difference disappears and only reappears at the would-be verb of the local coherence (see Supplementary Materials at https://osf.io/f6ntk). We speculate that the effect may be due to *einer*, “one,” being semantically more specific than *einige*, “some.”^10^ Our preregistration also contained a prediction for additional targeted regressions from the object region to the subject region in locally coherent sentences, due to participants checking agreement between the subject and the would-be verb. This prediction was not borne out either.^11^ Note, however, that this interpretation may be at odds with the findings of Gibson et al. (2013), where participants had ample opportunity to reread sentences and still gave interpretations that were inconsistent with the literal input. By contrast, it is not at odds with the findings of Levy (2011), where downstream effects of possible local edits were observed, as the self-paced reading paradigm did not allow readers to overtly regress.^12^ For further reading on how interactions can be recast as main effects, see Vasishth (2018) and Westfall (2015).^13^ Further information about the experiment can be found at https://osf.io/f6ntk.

## Supplemental Materials

https://osf.io/ersxn

https://osf.io/f6ntk

## References

[bib1] Altmann, G. T. (2011). Language can mediate eye movement control within 100 milliseconds, regardless of whether there is anything to move the eyes to. Acta Psychologica, 137(2), 190–200. **DOI:** https://doi.org/10.1016/j.actpsy.2010.09.009, **PMID:** 20965479 20965479 10.1016/j.actpsy.2010.09.009 PMC3118831

[bib2] Bever, T. G. (1970). The cognitive basis for linguistic structure. In J. R. Hayes (Ed.), Cognitive development of language (pp. 279–352). Wiley.

[bib3] Bicknell, K., Levy, R., & Demberg, V. (2009). Correcting the incorrect: Local coherence effects modeled with prior belief update. Proceedings of the Annual Meeting of the Berkeley Linguistics Society, 35(1), 13–24. 10.3765/bls.v35i1.3594

[bib4] Brants, S., Dipper, S., Hansen, S., Lezius, W., & Smith, G. (2002). The TIGER treebank. Proceedings of the Workshop on Treebanks and Linguistic Theories, 168, 24–41.

[bib5] Bürkner, P.-C. (2017). brms: An R package for Bayesian multilevel models using Stan. Journal of Statistical Software, 80(1), 1–28. 10.18637/jss.v080.i01

[bib6] Christianson, K., Hollingworth, A., Halliwell, J. F., & Ferreira, F. (2001). Thematic roles assigned along the garden path linger. Cognitive Psychology, 42(4), 368–407. **DOI:** https://doi.org/10.1006/cogp.2001.0752, **PMID:** 11368528 11368528 10.1006/cogp.2001.0752

[bib7] Christianson, K., Luke, S. G., Hussey, E. K., & Wochna, K. L. (2017). Why reread? Evidence from garden-path and local coherence structures. The Quarterly Journal of Experimental Psychology, 70(7), 1380–1405. **DOI:** https://doi.org/10.1080/17470218.2016.1186200, **PMID:** 27150840 27150840 10.1080/17470218.2016.1186200

[bib8] Eberhard, K. M. (1997). The marked effect of number on subject–verb agreement. Journal of Memory and Language, 36(2), 147–164. 10.1006/jmla.1996.2484

[bib9] Engbert, R., & Mergenthaler, K. (2006). Microsaccades are triggered by low retinal image slip. Proceedings of the National Academy of Sciences, 103(18), 7192–7197. **DOI:** https://doi.org/10.1073/pnas.0509557103, **PMID:** 16632611 10.1073/pnas.0509557103 PMC1459039 16632611

[bib10] Ferreira, F., Bailey, K. G., & Ferraro, V. (2002). Good-enough representations in language comprehension. Current Directions in Psychological Science, 11(1), 11–15. 10.1111/1467-8721.00158

[bib11] Frazier, L., & Rayner, K. (1982). Making and correcting errors during sentence comprehension: Eye movements in the analysis of structurally ambiguous sentences. Cognitive Psychology, 14(2), 178–210. 10.1016/0010-0285(82)90008-1

[bib12] Futrell, R., Gibson, E., & Levy, R. P. (2020). Lossy-context surprisal: An information-theoretic model of memory effects in sentence processing. Cognitive Science, 44(3), Article e12814. **DOI:** https://doi.org/10.1111/cogs.12814, **PMID:** 32100918 32100918 10.1111/cogs.12814 PMC7065005

[bib13] Gibson, E. (2006). The interaction of top–down and bottom–up statistics in the resolution of syntactic category ambiguity. Journal of Memory and Language, 54(3), 363–388. 10.1016/j.jml.2005.12.005

[bib14] Gibson, E., Bergen, L., & Piantadosi, S. T. (2013). Rational integration of noisy evidence and prior semantic expectations in sentence interpretation. Proceedings of the National Academy of Sciences, 110(20), 8051–8056. **DOI:** https://doi.org/10.1073/pnas.1216438110, **PMID:** 23637344 10.1073/pnas.1216438110 PMC3657782 23637344

[bib15] Hale, J. T. (2001). A probabilistic Earley parser as a psycholinguistic model. Proceedings of the Second Meeting of the North American Chapter of the Association for Computational Linguistics on Language Technologies, 103(18), 7192–7197. 10.3115/1073336.1073357

[bib16] Hale, J. T. (2011). What a rational parser would do. Cognitive Science, 35(3), 399–443. 10.1111/j.1551-6709.2010.01145.x

[bib17] Jeffreys, H. (1998). The theory of probability. Oxford University Press. (Original work published 1939)

[bib18] Konieczny, L. (2005). The psychological reality of local coherences in sentence processing. In B. G. Bara, L. W. Barsalou, & M. Bucciarelli (Eds.), Proceedings of the 27th Annual Conference of the Cognitive Science Society (pp. 1178–1183). Cognitive Science Society.

[bib19] Konieczny, L., Müller, D., Hachmann, W., Schwarzkopf, S., & Wolfer, S. (2009). Local syntactic coherence interpretation: Evidence from a visual world study. In N. A. Taatgen & H. van Rijn (Eds.), Proceedings of the 31st Annual Conference of the Cognitive Science Society (pp. 1133–1138). Cognitive Science Society.

[bib20] Kukona, A., Cho, P. W., Magnuson, J. S., & Tabor, W. (2014). Lexical interference effects in sentence processing: Evidence from the visual world paradigm and self-organizing models. Journal of Experimental Psychology: Learning, Memory, and Cognition, 40(2), 326–347. **DOI:** https://doi.org/10.1037/a0034903, **PMID:** 24245535 10.1037/a0034903 PMC4033295 24245535

[bib21] Lee, M. D., & Wagenmakers, E.-J. (2014). Bayesian cognitive modeling: A practical course. Cambridge University Press.

[bib22] Levy, R. (2008a). Expectation-based syntactic comprehension. Cognition, 106(3), 1126–1177. **DOI:** https://doi.org/10.1016/j.cognition.2007.05.006, **PMID:** 17662975 17662975 10.1016/j.cognition.2007.05.006

[bib23] Levy, R. (2008b). A noisy-channel model of human sentence comprehension under uncertain input. In M. Lapata & H. T. Ng (Eds.), Proceedings of the 2008 Conference on Empirical Methods in Natural Language Processing (pp. 234–243). Association for Computational Linguistics. 10.3115/1613715.1613749

[bib24] Levy, R. (2011). Integrating surprisal and uncertain-input models in online sentence comprehension: Formal techniques and empirical results. In A. Way & P. Pantel (Eds.), Proceedings of the 49th Annual Meeting of the Association for Computational Linguistics: Human Language Technologies (pp. 1055–1065). Association for Computational Linguistics.

[bib25] Levy, R., Bicknell, K., Slattery, T., & Rayner, K. (2009). Eye movement evidence that readers maintain and act on uncertainty about past linguistic input. Proceedings of the National Academy of Sciences, 106(50), 21086–21090. **DOI:** https://doi.org/10.1073/pnas.0907664106, **PMID:** 19965371 10.1073/pnas.0907664106 PMC2795489 19965371

[bib26] Lewandowski, D., Kurowicka, D., & Joe, H. (2009). Generating random correlation matrices based on vines and extended onion method. Journal of Multivariate Analysis, 100(9), 1989–2001. 10.1016/j.jmva.2009.04.008

[bib27] Logačev, P., & Vasishth, S. (2013). em2: A package for computing reading time measures for psycholinguistics [Computer software manual] (R package version 0.9). https://CRAN.R-project.org/package=em2. CRAN-R.

[bib28] McKoon, G., & Ratcliff, R. (2003). Meaning through syntax: Language comprehension and the reduced relative clause construction. Psychological Review, 110(3), 490–525. **DOI:** https://doi.org/10.1037/0033-295X.110.3.490, **PMID:** 12885112 12885112 10.1037/0033-295X.110.3.490 PMC1403829

[bib29] Meng, X.-L., & Wong, W. H. (1996). Simulating ratios of normalizing constants via a simple identity: A theoretical exploration. Statistica Sinica, 6, 831–860.

[bib30] Meseguer, E., Carreiras, M., & Clifton, C. (2002). Overt reanalysis strategies and eye movements during the reading of mild garden path sentences. Memory & Cognition, 30(4), 551–561. **DOI:** https://doi.org/10.3758/BF03194956, **PMID:** 12184556 12184556 10.3758/bf03194956

[bib31] Mitchell, D. C., Shen, X., Green, M. J., & Hodgson, T. L. (2008). Accounting for regressive eye-movements in models of sentence processing: A reappraisal of the selective reanalysis hypothesis. Journal of Memory and Language, 59(3), 266–293. 10.1016/j.jml.2008.06.002

[bib32] Morgan, E., Keller, F., & Steedman, M. (2010). A bottom-up parsing model of local coherence effects. In S. Ohlsson & R. Catrambone (Eds.), Proceedings of the Annual Meeting of the Cognitive Science Society (pp. 1559–1564).

[bib33] Müller, H., & Konieczny, L. (2019, March 29–31). The effect of context on local syntactic coherence processing [Poster]. 32nd Annual CUNY Conference on Human Sentence Processing. University of Colorado Boulder.

[bib34] Paape, D., & Vasishth, S. (2016). Local coherence and preemptive digging-in effects in German. Language and Speech, 59(3), 387–403. **DOI:** https://doi.org/10.1177/0023830915608410, **PMID:** 29924534 29924534 10.1177/0023830915608410

[bib35] R Core Team. (2019). R: A language and environment for statistical computing [Computer software manual]. https://www.R-project.org.

[bib36] Raftery, A. E. (1995). Bayesian model selection in social research. Sociological Methodology, 25, 111–163. 10.2307/271063

[bib37] Rayner, K. (2009). Eye movements and attention in reading, scene perception, and visual search. The Quarterly Journal of Experimental Psychology, 62(8), 1457–1506. **DOI:** https://doi.org/10.1080/17470210902816461, **PMID:** 19449261 19449261 10.1080/17470210902816461

[bib38] Rouder, J. N. (2005). Are unshifted distributional models appropriate for response time? Psychometrika, 70(2), 377–381. 10.1007/s11336-005-1297-7

[bib39] Smith, G. (2018). A theory of timing effects in a self-organizing model of sentence processing (Unpublished doctoral dissertation). University of Connecticut.

[bib40] Smith, G., Franck, J., & Tabor, W. (2018). A self-organizing approach to subject–verb number agreement. Cognitive Science, 42(S4), 1043–1074. **DOI:** https://doi.org/10.1111/cogs.12591, **PMID:** 29388256 29388256 10.1111/cogs.12591

[bib41] Sturt, P., Pickering, M. J., & Crocker, M. W. (1999). Structural change and reanalysis difficulty in language comprehension. Journal of Memory and Language, 40(1), 136–150. 10.1006/jmla.1998.2606

[bib42] Tabor, W., Galantucci, B., & Richardson, D. (2004). Effects of merely local syntactic coherence on sentence processing. Journal of Memory and Language, 50(4), 355–370. 10.1016/j.jml.2004.01.001

[bib43] Tabor, W., & Hutchins, S. (2004). Evidence for self-organized sentence processing: Digging-in effects. Journal of Experimental Psychology: Learning, Memory, and Cognition, 30(2), 431–450. **DOI:** https://doi.org/10.1037/0278-7393.30.2.431, **PMID:** 14979816 10.1037/0278-7393.30.2.431 14979816

[bib44] van Schijndel, M., & Linzen, T. (2018). Modeling garden path effects without explicit hierarchical syntax. In T. Rogers, M. Rau, J. Zhu, & C. Kalish (Eds.), Proceedings of the 40th Annual Conference of the Cognitive Science Society (pp. 2600–2605). Cognitive Science Society.

[bib45] Vasishth, S. (2018, April 8). The relationship between paired t-tests and linear mixed models in a 2 × 2 repeated measures design. https://vasishth-statistics.blogspot.com/2018/04/the-relationship-between-paired-t-tests.html.

[bib46] Villata, S., & Franck, J. (2020). Similarity-based interference in agreement comprehension and production: Evidence from object agreement. Journal of Experimental Psychology: Learning, Memory, and Cognition, 46(1), 170–188. **DOI:** https://doi.org/10.1037/xlm0000718, **PMID:** 31033310 10.1037/xlm0000718 31033310

[bib47] von der Malsburg, T., & Vasishth, S. (2011). What is the scanpath signature of syntactic reanalysis? Journal of Memory and Language, 65(2), 109–127. 10.1016/j.jml.2011.02.004

[bib48] von der Malsburg, T., & Vasishth, S. (2013). Scanpaths reveal syntactic underspecification and reanalysis strategies. Language and Cognitive Processes, 28(10), 1545–1578. 10.1080/01690965.2012.728232

[bib49] Vosse, T., & Kempen, G. (2000). Syntactic structure assembly in human parsing: A computational model based on competitive inhibition and a lexicalist grammar. Cognition, 75(2), 105–143. **DOI:** https://doi.org/10.1016/S0010-0277(00)00063-9, **PMID:** 10771275 10771275 10.1016/s0010-0277(00)00063-9

[bib50] Westfall, J. (2015). Follow-up: What about Uri’s 2n rule? https://jakewestfall.org/blog/index.php/2015/05/27/follow-up-what-about-uris-2n-rule.

